# Ocular surface microbiota alterations in patients with pterygium

**DOI:** 10.3389/fcimb.2025.1647973

**Published:** 2026-01-16

**Authors:** Yiyuan Guo, Gege Tian, Guangzhong Feng, Yong Li, Biying Wang, Jiaxin Wang, Hong Zhang, Yongsheng Hou

**Affiliations:** 1 Department of Ophthalmology, The First Affiliated Hospital of Harbin Medical University, Harbin, China; 2 College of Arts and Sciences, Northeast Agricultural University, Harbin, China; 3 Bioinformatics Center, Northeast Agricultural University, Harbin, China

**Keywords:** 16S rRNA sequencing, microbial community, ocular surface microbiota, pathogenesis, pterygium

## Abstract

**Purpose:**

To examine the alterations in the ocular surface microbiota and microbial diversity in patients with pterygium after different durations of electronic device use.

**Methods:**

This study involved 31 individuals diagnosed with unilateral pterygium. Conjunctival sac swabs were collected from both eyes, and 16S rRNA sequencing was used to identify the species and quantity of bacteria. The microbial composition was annotated and represented through comprehensive bioinformatics analysis.

**Results:**

The alpha diversity did not differ significantly between the eyes with pterygium and the contralateral eyes. The Chao1 and Shannon indices for the eyes with pterygium of the patients who used electronic devices for extended periods were significantly higher than those for their contralateral eyes. Principal coordinate analysis revealed that the beta diversity of the eyes with pterygium was similar to that of the contralateral eyes. Genus-level differential analysis revealed that the relative abundance of *Pseudomonas* was significantly increased and that of *Bacteroides* was significantly decreased in the eyes with pterygium. The relative abundance of *Comamonas* was significantly higher in the eyes with pterygium than in the contralateral eyes of the patients who used electronic devices for more than 4 h per day.

**Conclusion:**

The ocular surface of eyes with pterygium had increased colonization by opportunistic pathogenic bacteria. Excessive exposure to blue light, which may result from prolonged use of electronic devices, may be a risk factor for the development of pterygium.

## 1 Introduction

Pterygium is a highly prevalent conjunctival disorder characterized by chronic degenerative inflammatory lesions. It presents as a triangular fibrovascular proliferation consisting of conjunctival epithelium and proliferative subconjunctival tissue. Typically, this lesion originates from the nasal aspect of the palpebral fissure and extends toward the corneal surface (Shen et al., 2020). Clinically, pterygium often manifests with ocular redness, irritation, and dryness (Hussain et al., 2020). In severe cases, corneal involvement may progress to the pupillary region, resulting in visual impairment (Li et al., 2022). Ultraviolet radiation, viral infection, and aging have been recognized as key risk factors (Palewski et al., 2022). Surgical excision remains the primary treatment modality; however, postoperative recurrence poses a significant clinical challenge (Hacıoğlu and Erdöl, 2017). Significant progress has been made in research on the human microbiome. Previous studies have demonstrated that alterations in the microbial populations of the skin, vagina, oral cavity, and stomach can have a major impact on body homeostasis (Tozzo et al., 2022). In recent years, the potential role of the ocular surface microbiota in health and disease has gradually attracted attention. The significance of the ocular surface microbiota in disorders such as blepharitis, meibomian gland dysfunction, keratitis, and dry eye disease has previously been confirmed by previous studies (Yang et al., 2022). However, research focusing on the ocular surface microbiota in the context of pterygium remains limited. Moreover, the association between changes in the ocular surface microbiota and the development of pterygium has not yet been fully elucidated. Given the critical regulatory role of the microbiome plays in preserving ocular immune homeostasis, this study was designed to evaluate the ocular surface microbiota in patients with pterygium. We used 16S rRNA sequencing to analyze the microbial composition and diversity of eyes with pterygium and their contralateral eyes to characterize the colonization patterns of the ocular surface microbiota colonization patterns.

## 2 Methods and materials

The study adhered to the principles of the Declaration of Helsinki, and all participants provided written informed consent. The study protocol was approved by the Ethics Committee of the First Affiliated Hospital of Harbin Medical University approved the study protocol (MR-23-25-075763).

### 2.1 Participants

A cohort of 31 patients diagnosed with unilateral pterygium was recruited from the Eye Department of the First Affiliated Hospital of Harbin Medical University between November 2023 and February 2024. A comprehensive medical history was obtained from each patient, followed by a series of ophthalmic examinations. Based on the assessment results, patients were grouped into the pterygium-affected eye group (PE group), which included the eyes with pterygium from all 31 participants, and the contralateral eye group (CE group), which included the contralateral healthy eyes from 26 participants. The inclusion criterion was a confirmed diagnosis of unilateral primary pterygium. The exclusion criteria were as follows: having acute ocular inflammation within the previous month; receiving systemic antibiotic therapy within the past 3 months; use of medications that could affect the ocular microbial community, such as antibiotics or corticosteroids, within the past month; and having systemic diseases affecting immune function or ocular health.

### 2.2 Sample collection

Microbiological samples were collected from the conjunctiva of each patient using disposable sterile dry swabs. The swabs were gently moved across the inferior bulbar conjunctiva 2–3 times with uniform pressure. After each collection, a sterile swab was used as a blank control after each collection to ensure sample quality and reliability. The swabs were placed in sterile containers, transported on ice to the laboratory, and stored at −80°C for testing and DNA extraction and analysis.

All samples were collected by the same ophthalmologist in an ultraviolet-sterilized ocular examination room while wearing a sterile masks and gloves. The same sampling procedure was used for both eyes to ensure consistency.

### 2.3 DNA extraction and amplification

Genomic DNA was extracted from the collected samples using the MagPure Soil DNA LQ Kit (Magan, China) according to the manufacturer’s instructions. The concentration and purity of the extracted DNA were assessed by agarose gel electrophoresis and using a NanoDrop 2000 spectrophotometer (Thermo Fisher Scientific, USA). The purified DNA was stored at −20 °C until further use.

The extracted DNA was used as a template for PCR amplification of the bacterial 16S rRNA gene using specific barcoded primers with barcodes and Takara Ex Taq high-fidelity DNA polymerase (Takara, Japan). The V3–V4 variable regions of the 16S rRNA gene were amplified for bacterial diversity analysis using the universal primers 343F (5’-TACGGRAGGCAGCAG-3’) and 798R (5’-AGGGTATCTAATCCT-3’).

### 2.4 Library preparation and sequencing

Agarose gel electrophoresis was used to verify the PCR amplification products. The amplicons were purified using AMPure XP magnetic beads and used as templates for the subsequent PCR amplification cycle. After a second purification using magnetic beads, the purified PCR products were quantified using a Qubit fluorometer (Thermo Fisher Scientific, USA), and their concentrations were adjusted for sequencing. Sequencing was performed using the Illumina NovaSeq 6000 platform (Illumina, USA) to generate 250 bp paired-end reads. All sequencing procedures were performed by OE Biotech Co., Ltd. (Shanghai, China).

### 2.5 Bioinformatics analysis

OE Biotech Co., Ltd. (Shanghai, China) performed data processing, sequencing, and library construction. Raw sequencing data were generated in FASTQ format. Primer sequences were removed from the raw data using the Cutadapt program. The qualified paired-end reads were subsequently processed using the DADA2 (Callahan et al., 2016) plugin in QIIME2 (Bolyen et al., 2019) (version 2020.11) for quality filtering, denoising, merging, and chimera removal to obtain representative sequences and an amplicon sequence variant (ASV) abundance table.

The QIIME2 pipeline was used to select representative sequences for each ASV, which were taxonomically classified using the q2-feature-classifier plugin against the SILVA database (version 138). Alpha diversity was assessed using the Chao1 and Shannon indices. Principal coordinate analysis was performed using R software to evaluate differences between the samples, and beta diversity was determined using the unweighted UniFrac distance matrix. Linear discriminant analysis effect size (LEfSe) software (version 1.0) was used to identify putative microbial biomarkers based on linear discriminant analysis (LDA) scores of > 2.

### 2.6 Statistical analysis

Statistical analyses were performed using SPSS software (version 23.0; IBM Corp., Armonk, NY, USA). Categorical data were presented as proportions, and continuous variables were presented as the mean ± standard deviation. Microbial genus-level differences between the two groups were analyzed using the t-test. The Mann–Whitney U test and one-way analysis of variance were used to determine the differences among the groups. Significant variations in community structure across the groups were also determined using permutational multivariate analysis of variance based on distance matrices. Statistical significance was set at p < 0.05.

## 3 Results

### 3.1 Patient characteristics and sequencing quality control

Thirty-one individuals with unilateral pterygium were invited to participate in the study.

**Figure 1:**
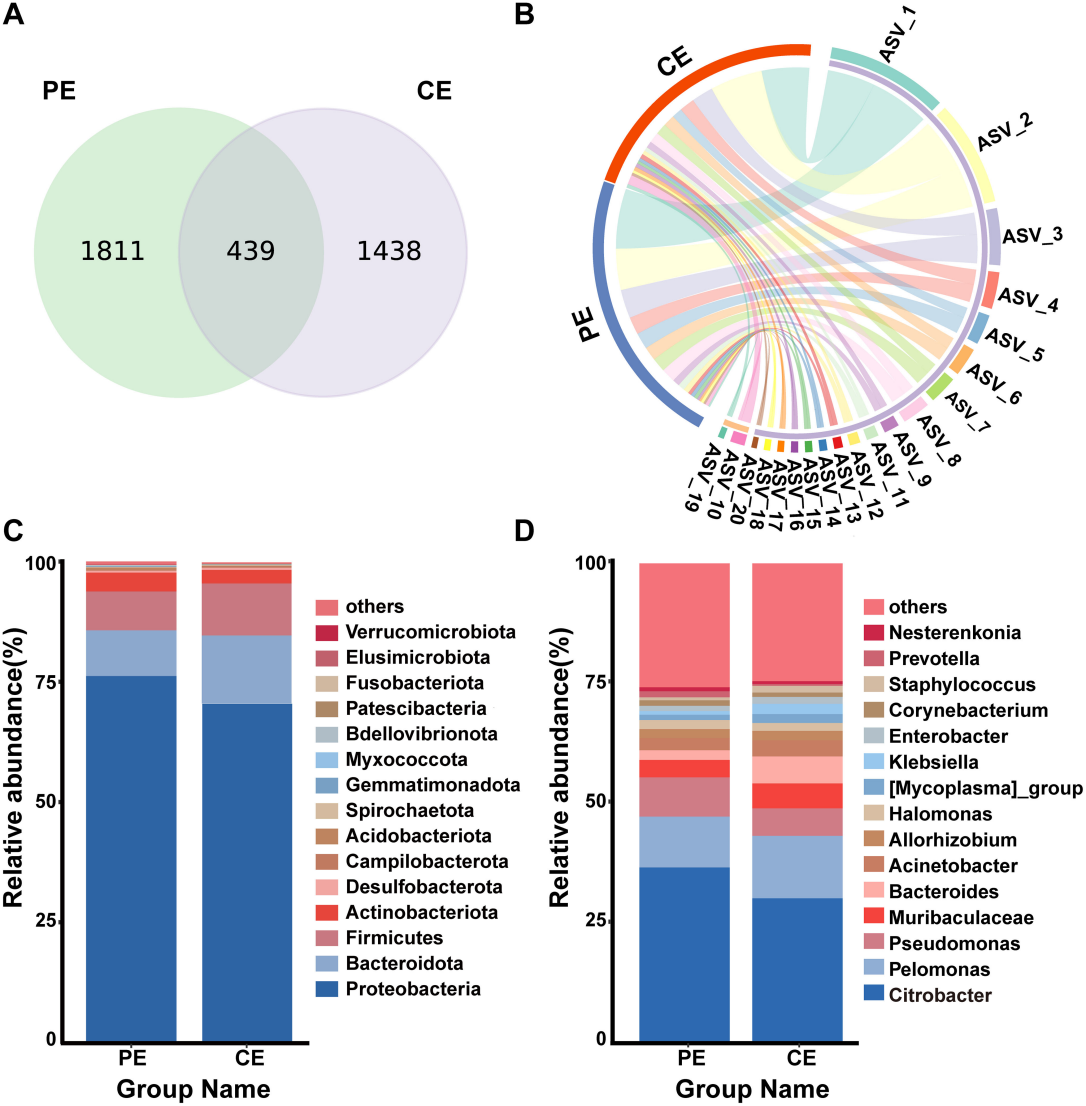
**(A)** Venn diagram showing the overlap and differences in ASVs between PE group and CE group. **(B)** Circlize plot of ASVs of PE group and CE group. **(C)** Differences in relative mean abundances of phylum in ocular microbiota between the PE and CE groups. **(D)** Differences in relative mean abundances of genus in ocular microbiota between the PE and CE groups.

**Table 1** provides a comprehensive summary of their demographic characteristics. Their ages ranged from 36 to 76 years, with a mean of 58.6 ± 9.4 years. Seventeen (54.8%) and 14 (45.2%) were female and male, respectively. The mean tear film breakup time (TBUT) of the pterygium-affected eyes (8.0 ± 1.8 s) was significantly lower than that of the contralateral eyes (10.7 ± 1.9 s) (P < 0.001; **Table 2**). The 16S rRNA sequencing yielded 78,015–81,955 raw reads per sample. The number of clean tags after quality control was 68,463–76,672. The number of valid tags after the elimination of chimeras was 65,109–76,272.The number of ASVs per sample ranged from 76 to 252. Venn diagram analysis revealed 1,811 and 1,438 unique ASVs in the PE and CE groups, respectively. They shared 439 ASVs (**Figure 1A**). The ASVs of the PE and CE groups were significantly different (exact binomial test, p < 2.2×10^-16^; **Figure 1B**).

**Table 1:** Demographic characteristics of the patients with pterygium.

Features	Patients with pterygium (n=31)
Mean ± SD	58.6 ± 9.4
Range	36-76
Female, n (%)	17(54.8)
Male, n (%)	14(45.2)

SD, standard deviation.

**Table 2:** Tear breakup time of the groups.

	PE group n=31	CE group n=26	P-value
TBUT (s)			p<0.001
Mean ± SD	8.0 ± 1.8	10.7 ± 1.9	
Range	5-13	7-14	

TBUT, Tear breakup time.

### 3.2 Species annotation analysis

The ASV sequences were taxonomically annotated to evaluate variations in the ocular surface microbial composition between the contralateral and pterygium-affected and contralateral eyes. A total of 395 genera and 24 bacterial phyla were identified. At the phylum level, *Proteobacteria*, *Bacteroidota*, *Firmicutes*, and *Actinobacteriota* dominated the microbial communities of both groups, accounting for more than 80% of the total relative abundance (**Figure 1C**).

The relative abundance of Proteobacteria was slightly higher in the PE group (76.14%) than in the CE group (70.57%), whereas the relative abundance of Bacteroidota was lower in the PE group (9.54%) than in the CE group (14.20%). However, these differences were not statistically significant (*Bacteroidota*: p = 0.07; *Proteobacteria*: p = 0.13). The relative abundances of *Firmicutes* and *Actinobacteria* were 10.84% and 2.82%, respectively, in the CE group and 8.08% and 3.95%, respectively, in the PE group, showing comparable distributions between groups.

At the genus level, the most prevalent taxa in both groups were *Citrobacter*, *Pelomonas*, and *Pseudomonas*, accounting for 55.40% and 48.94% of the total relative abundance in the PE and CE groups, respectively (**Figure 1D**).

### 3.3 Ocular surface microbiota analysis for the control and PE groups

**Figure 2:**
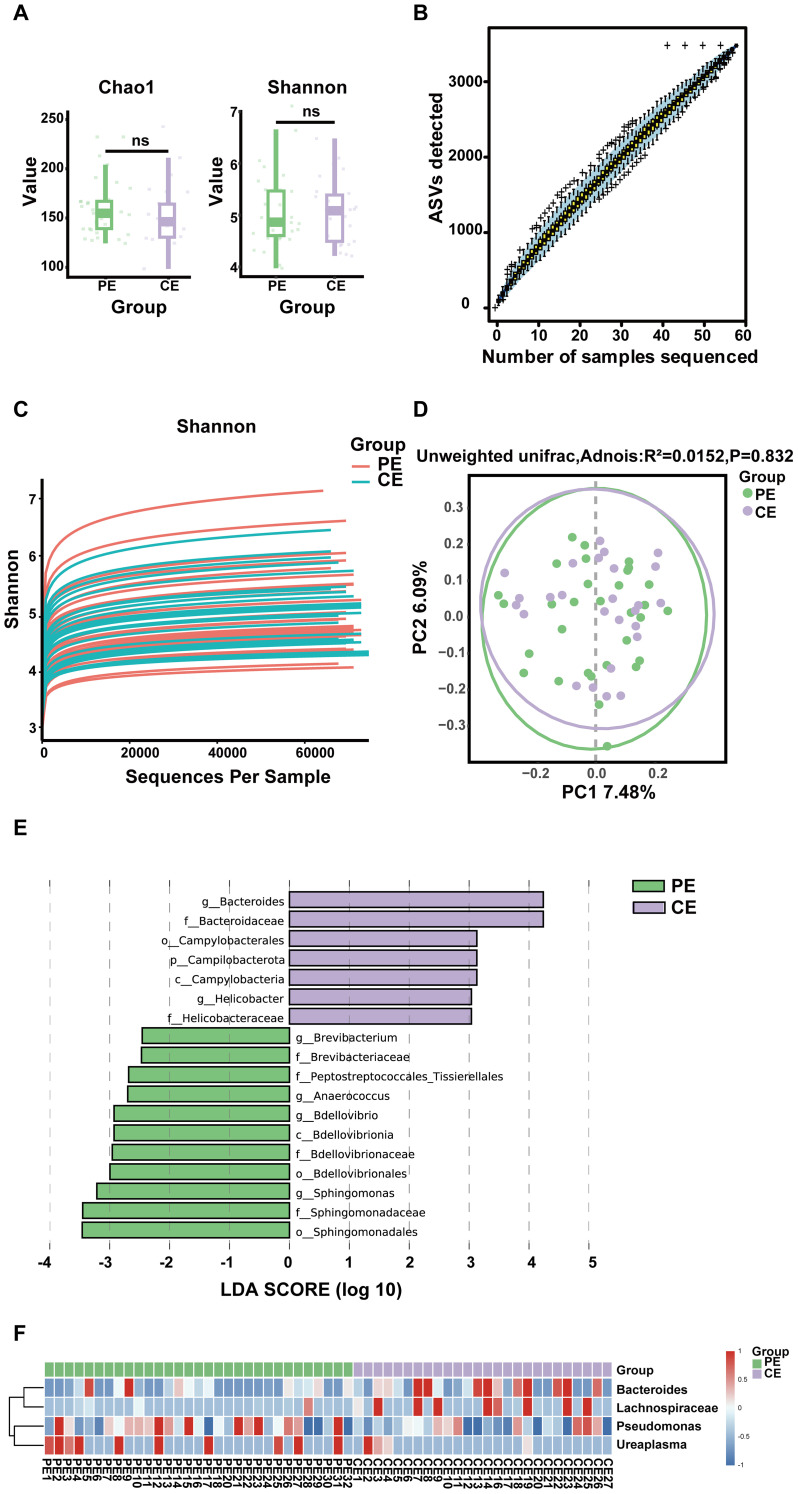
**(A)** Box plots of the Chao1 and Shannon indices show no significant differences in species diversity between the PE and CE groups. **(B)** Species accumulation boxplot indicates a progressive increase in observed ASVs with increasing sample size, approaching a plateau. **(C)** The Shannon index rarefaction curve suggests that the sequencing data are sufficient and additional data will not significantly affect the alpha diversity index. **(D)** Beta diversity analysis of the ocular surface microbiota in the PE and CE groups, as visualized using a PCoA plot (ns: not significant). **(E)** LEfSe analysis for the characteristic colony search between the PE and CE groups. **(F)** Heatmap of genus-level relative abundance cluster analysis of the PE and CE groups.

Alpha diversity reflects the richness and evenness of the microbial populations within a sample. The Chao1 and Shannon indices were used to assess the richness and evenness of the two sets of samples. The data showed no discernible difference in alpha diversity in both groups. No significant differences in alpha diversity were observed, indicating comparable microbial richness and evenness between the PE and CE groups (**Figure 2A**).

Species accumulation boxplots showed that the number of observed ASVs increased steadily with the increasing number of samples, approaching a plateau. This trend indicates that the sample size and approached a plateau, suggesting that the sample size was sufficient to capture the majority of the microbial diversity in the dataset (**Figure 2B**). The rarefaction curves similarly reached a plateau, indicating adequate sequencing depth and satisfactory species coverage was satisfactory. A larger amount of data would not significantly affect the alpha diversity index (**Figure 2C**).

Beta diversity analysis is shown in **Figure 2D** and illustrates variation in microbial community structure between the groups. The findings revealed no significant difference between the groups. No significant difference was detected (R² = 0.0152, p = 0.832), suggesting similar ocular surface microbiota profiles, likely due to the paired nature of the samples.

**Figure 3:**
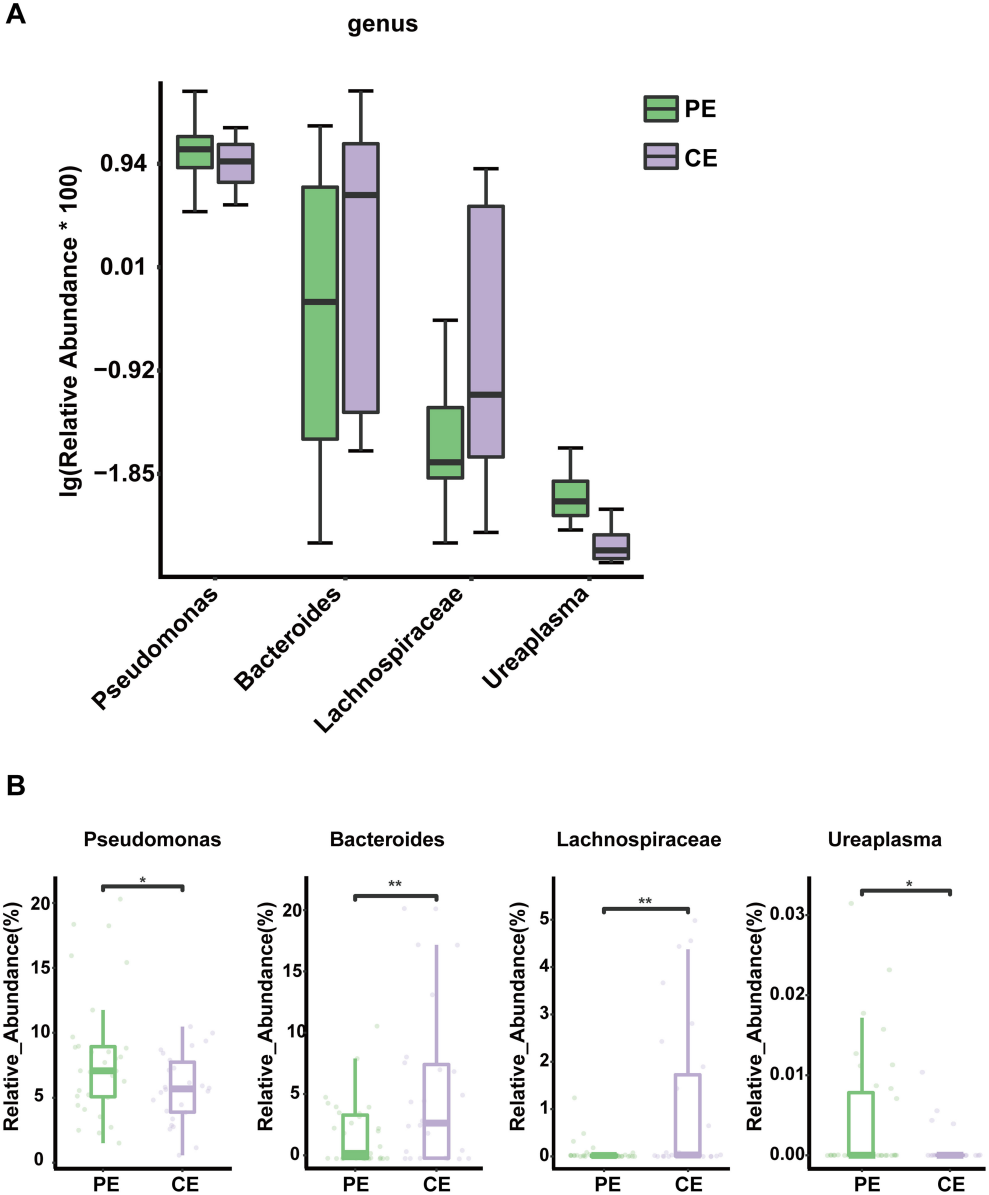
**(A)** Boxplot of the relative abundances of the dominant genera in the PE and CE groups. **(B)** Boxplots show the abundance distribution of individual genera across different groups. *P<0.05; **P<0.01.

LEfSe analysis identified potential microbial biomarkers associated with pterygium-affected and contralateral eyes. The LDA score distribution histogram revealed 18 significantly different biomarkers between the two groups (LDA score > 2, p < 0.05). Four genera—*Brevibacterium*, *Anaerococcus*, *Bdellovibrio*, and *Sphingomonas*—were significantly enriched in the PE group, whereas *Bacteroides* and *Helicobacter*, were significantly enriched in the CE group (**Figure 2E**). Genus-level differences are illustrated in **Figures 2F** and **3A**, with *Bacteroides* and *Lachnospiraceae* enriched in the CE group and *Pseudomonas* and *Ureaplasma* were enriched in the PE group (**Figure 3B**).

### 3.4 Ocular surface microbiota analysis based on electronic device usage duration in patients with pterygium

**Figure 4:**
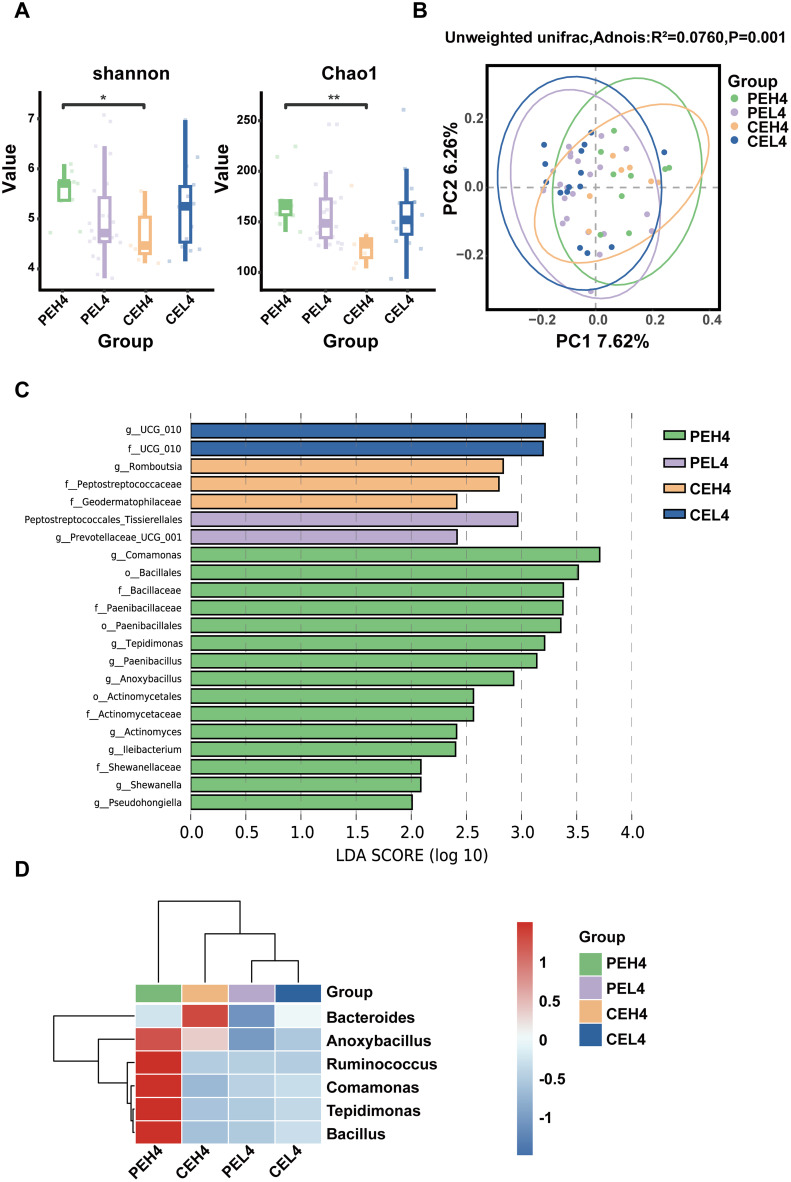
**(A)** Box plots of the Chao1 and Shannon indices showing significant differences in species diversity among the electronic device viewing durations of the patients with pterygium. **(B)** PCoA plot showing the beta diversity analysis for the different electronic device viewing durations of the patients with pterygium (*P<0.05; **P<0.01). **(C)** LEfSe analysis for the identification of characteristic colonies in the groups. **(D)** Heatmap of genus-level relative abundance cluster analysis for the groups.

To investigate the impact of electronic device usage duration on the ocular surface microbiota of the patients with pterygium, participants were categorized into four groups: PEH4 (pterygium-affected eyes, ≥4 h/d), CEH4 (contralateral eyes, ≥4 h/d), PEL4 (pterygium-affected eyes, <4 h/d), and CEL4 (contralateral eyes, <4 h/d). Alpha diversity analysis showed a significant increase in microbial diversity in the PEH4 group compared with the CEH4 group (**Figure 4A**), indicating that prolonged electronic device use was associated with increased microbial diversity specifically in the pterygium-affected eyes. Beta diversity analysis revealed significant differences in microbial community structures among the four groups (PERMANOVA, p = 0.001; **Figure 4B**).

**Figure 5:**
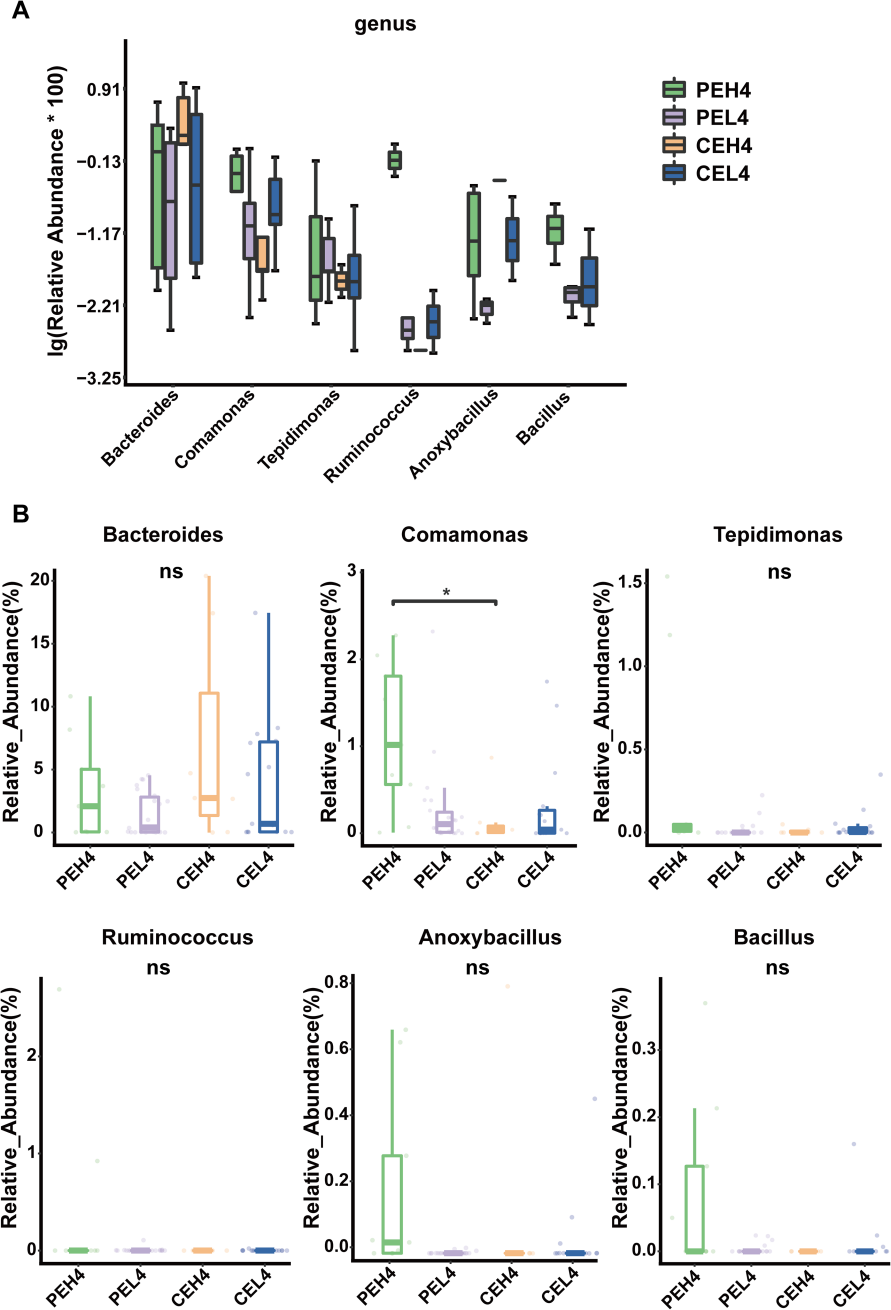
**(A)** Boxplot of the relative abundances of the dominant genera in the groups. **(B)** Boxplots show the abundance distribution of individual genera across different groups. *P<0.05; ns, not significant.

LEfSe analysis was performed to further assess the influence of the duration of electronic device usage duration on the potential microbial biomarkers in patients with pterygium. Eight genera, including *Tepidimonas*, *Paenibacillus*, *Anoxybacillus*, *Actinomyces*, *Ileibacterium*, *Shewanella*, and *Pseudohongiella*, were significantly enriched in the PEH4 group. One genus, *Romboutsia*, was significantly enriched in the CEH4 group (**Figure 4C**). In addition, genus-level differences are presented in **Figures 4D** and **5A**, the relative abundance of Comamonas was significantly higher in the PEH4 group than in the CEH4 group (**Figure 5B**).

## 4 Discussion

The microbiome, an essential component of the ocular surface microenvironment, is influenced by host factors, environmental exposures, and iatrogenic interventions and plays a key role in local and systemic immune responses (Morgan et al., 2013; Doan et al., 2016). Ocular surface microbiota contributes to ocular disorders by disrupting immunological homeostasis, altering local immune function, and causing chronic inflammation (Zysset-Burri et al., 2021). The ocular surface microbiota of pterygium-affected eyes and contralateral eyes was examined. Proteobacteria, Bacteroidota, Firmicutes, and Actinobacteriota accounted for the majority of the ASVs detected in the conjunctival microbial samples from both groups. Findings from previous studies of the ocular surface microbiota (Huang et al., 2016; Gomes et al., 2020; Zilliox et al., 2020) suggest that the microbial composition observed in this study is typical.

No statistically significant differences in overall ocular surface microbial diversity were observed between pterygium-affected eyes and contralateral eyes. However, the pterygium-affected eyes with longer electronic device usage durations exhibited significantly higher microbial richness than the contralateral eyes. These findings suggest that prolonged electronic device use may be a potential risk factor for the development of pterygium. Blue light is present in many artificial light sources, including the screens of electronic devices such as mobile phones, computers, tablets, and televisions. Blue light has relatively high energy within the visible spectrum and a wavelength range close to that of ultraviolet radiation (Stamatacos and Harrison, 2014), which is a well-established risk factor for pterygium. Although both eyes of an individual are typically exposed to similar levels of ultraviolet radiation, pterygium often develops unilaterally (Ishioka et al., 2001). The detrimental effects of ultraviolet exposure on the ocular surface have been unequivocally demonstrated in previous studies. Ultraviolet radiation has been reported to modulate the ocular surface microbiota and alter antimicrobial peptide expression, thereby disrupting the healthy balance between the microbiome and ocular surface immunity and promoting chronic ocular surface inflammation (Patra et al., 2018; Shibata et al., 2020). Blue light exposure has also been shown to induce epithelial–mesenchymal transition (EMT) in conjunctival epithelial cells, promote collagen deposition, and enhance cell migration (Yang et al., 2025). EMT and collagen deposition serve as key mechanisms driving lesion progression and corneal invasion (Meshkani et al., 2019). Pterygium is a degenerative conjunctival disorder of the conjunctiva characterized by corneal invasion and collagen deposition (Chui et al., 2008; Martín-López et al., 2021). Degeneration and dysfunction of the conjunctival tissue form the pathological basis of this disease. In the present study, the relative abundance of *Comamonas* increased in pterygium-affected eyes with longer electronic device usage durations. *Comamonas* primarily originates from natural environments, such as aquatic habitats and soil (Hem et al., 2022). It is recognized as an opportunistic human pathogen. Cases of corneal infection caused by Comamonas acidovorans have been reported (Lee et al., 2008; Wang et al., 2023). The virulence factors of the Comamonas genus are highly diverse (Wu et al., 2018). Blue light exposure may alter the ocular surface microenvironment, thereby creating favorable conditions for *Comamonas* colonization. These findings suggest that excessive blue light exposure may contribute to the pathogenesis and progression of pterygium, potentially through ocular surface dysbiosis and subsequent chronic inflammation.

Recent studies have reported a close association between ocular surface dysbiosis and pterygium pathogenesis of pterygium (Willis et al., 2020; Chang and Winn, 2023). The relative abundance of *Pseudomonas* was significantly higher in the pterygium-affected eyes, whereas that of *Bacteroides* was significantly lower. *Pseudomonas* has been detected in samples from the fornix and conjunctival tissues of patients with pterygium (Ozkan et al., 2018). This is consistent with our findings and supports their reliability. *Pseudomonas* is an opportunistic pathogen closely associated with inflammation (Huang et al., 2022). It possesses flexible metabolic capabilities that enable colonization across diverse environmental niches (De Meyer and Carlier, 2023). *Pseudomonas aeruginosa* can cause severe corneal diseases (Gadjeva et al., 2010). The biofilms formed by this bacterium and the various virulence factors it secretes, such as elastase and exotoxin A, play critical roles in its pathogenicity (Gupta et al., 2016; Sultan et al., 2021; Kaya et al., 2022). In this study, tear film breakup time was significantly shorter in pterygium-affected eyes. Previous studies have reported an association between pterygium formation and reduced tear film stability, suggesting that tear film dysfunction may be involved in disease development of pterygium (Ishioka et al., 2001). The ocular surface tear film contains various substances, such as growth factors, antimicrobial peptides, secretory IgA, and vitamins, that maintain ocular surface health by supporting immune homeostasis and host defense (Ponzini et al., 2021; Yoon et al., 2022). The increased colonization of opportunistic pathogens in the eyes with pterygium-affected eyes may therefore be attributable to impaired tear film stability, which weakens the ocular surface immune barrier and alters the ocular surface inflammatory state, thereby contributing to disease progression. *Bacteroides* species are rarely detected on the ocular surface using conventional culture methods (Parker et al., 2019). *Bacteroides*, a common Gram-negative anaerobes of the human cecum and colon, *Bacteroides* play a key role in maintaining intestinal barrier integrity (Pascal et al., 2017; Nomura et al., 2021; Gul et al., 2022). Its reduced abundance on the ocular surface may reflect diminished local defense capacity. The colonization of non-resident bacteria on the ocular surface observed in this study may be associated with mucosal damage and subsequent microbial penetration of *Bacteroides* into the submucosal tissue and the resulting infection through damaged mucosa (Wang et al., 2021). In summary, ocular surface dysbiosis in eyes affected by pterygium-affected eyes may promote chronic inflammation and contribute to disease onset and progression. Probiotics have been proposed as a potential strategy for regulating ocular surface immunity and alleviating chronic ocular inflammation (Yamazaki et al., 2020; Layús et al., 2021). Future research may focus on the local application of antimicrobial peptide eye drops and on reducing the duration of electronic device usage duration to inhibit the overgrowth of opportunistic pathogens. These strategies may help improve the ocular surface microbiota composition and tear film function and contribute to the prevention and management of pterygium and other ocular surface immune-inflammatory diseases.

This study has several limitations. First, the sample size was relatively small, which may limit the generalizability of the findings. Larger cohort studies involving larger cohorts are required to validate these results. Second, this study focused exclusively on the bacterial communities of the ocular surface in patients with pterygium and did not assess other microbial groups, such as fungi or viruses, limiting comprehensive characterization of ocular surface microecology. In addition, functional predictions were limited, making it difficult to elucidate the metabolic potential and underlying pathogenic mechanisms of the identified microorganisms. Future studies employing metagenomic sequencing may address these limitations and provide more comprehensive insights into the relationship between the ocular surface microbiome and pterygium. Finally, as this was a single-center study, regional and population-specific factors may have influenced the results. Multicenter studies involving diverse populations are needed to confirm the reliability and reproducibility of these findings.

In conclusion, patients diagnosed with pterygium exhibited distinct alterations in ocular surface bacterial colonization, characterized by increased abundance of opportunistic pathogens. Electronic device usage duration of electronic device use influenced ocular surface microbial diversity of the ocular surface in these patients. Prolonged exposure to blue light from electronic devices may represent a potential risk factor for pterygium, providing new theoretical insights into its pathogenesis.

## Data Availability

The datasets presented in this study can be found in the NCBI Sequence Read Archive, accession number PRJNA1279637.
