# Supplementary material for: Ocular surface microbiota alterations in patients with pterygium
Source: Front Cell Infect Microbiol. 2026 Jan 16;15:1647973. doi: 10.3389/fcimb.2025.1647973 (PMC12855565; doi:10.3389/fcimb.2025.1647973)
Supplement: Supplementary file 1 [file DataSheet1.pdf]

Supplementary Table 1

ANOSIM analysis of the groups.

		PE-CE
ANOSIM	R	0.04096
	P-value	0.055

PERMANOVA analysis of the groups.

Statistical test		PEH4-PEL4
PERMANOVA	R^2	0.0399
	P-value	0.001

R-value: between (−1, 1), R-value greater than 0 indicates that the difference between groups is greater than the difference within groups, R-value less than 0 indicates that the difference within groups is greater than the difference between groups; R:indicates the degree to which the sample variance is explained by different groups, i.e., the ratio of subgroup variance to total variance; the higher the R^2, the greater the degree to which the subgroups explain the variance; p-value: less than 0.05 means the test is highly reliable.
